# Abrogation of Plasminogen Activator Inhibitor-1-Vitronectin Interaction Ameliorates Acute Kidney Injury in Murine Endotoxemia

**DOI:** 10.1371/journal.pone.0120728

**Published:** 2015-03-23

**Authors:** Kamlesh K. Gupta, Deborah L. Donahue, Mayra J. Sandoval-Cooper, Francis J. Castellino, Victoria A. Ploplis

**Affiliations:** 1 W. M. Keck Center for Transgene Research, University of Notre Dame, Notre Dame, Indiana, United States of America; 2 Department of Chemistry and Biochemistry, University of Notre Dame, Notre Dame, Indiana, United States of America; University of Kentucky, UNITED STATES

## Abstract

Sepsis-induced acute kidney injury (AKI) contributes to the high mortality and morbidity in patients. Although the pathogenesis of AKI during sepsis is poorly understood, it is well accepted that plasminogen activator inhibitor-1 (PAI-1) and vitronectin (Vn) are involved in AKI. However, the functional cooperation between PAI-1 and Vn in septic AKI has not been completely elucidated. To address this issue, mice were utilized lacking either PAI-1 (PAI-1^−/−^) or expressing a PAI-1-mutant (PAI-1^R101A/Q123K^) in which the interaction between PAI-1 and Vn is abrogated, while other functions of PAI-1 are retained. It was found that both PAI-1^−/−^ and PAI-1^R101A/Q123K^ mice are associated with decreased renal dysfunction, apoptosis, inflammation, and ERK activation as compared to wild-type (WT) mice after LPS challenge. Also, PAI-1^−/−^ mice showed attenuated fibrin deposition in the kidneys. Furthermore, a lack of PAI-1 or PAI-1-Vn interaction was found to be associated with an increase in activated Protein C (aPC) in plasma. These results demonstrate that PAI-1, through its interaction with Vn, exerts multiple deleterious mechanisms to induce AKI. Therefore, targeting of the PAI-1-Vn interaction in kidney represents an appealing therapeutic strategy for the treatment of septic AKI by not only altering the fibrinolytic capacity but also regulating PC activity.

## Introduction

Sepsis initiated by gram-negative bacteria is often accompanied by acute kidney injury (AKI). Development of AKI during sepsis is associated with higher mortality, increased morbidity, and often results in multiple organ damage, and therefore is a major public health concern in patients [1–4]. Currently, no effective treatment is available for preventing life-threatening septic AKI, due to a general lack of mechanistic relationships between the inflammatory response, inflammatory signaling pathways, and end-organ failure. Therefore, a clearer understanding of the cellular and molecular mechanisms of the processes involved in the development of septic AKI will facilitate in uncovering new therapeutic approaches.

The hemostasis system is a well-established target for bacterial toxins, *e*.*g*., lipopolysaccharide (LPS), and alterations in the activation of procoagulant and fibrinolytic systems together with strong proinflammatory responses are thought to play significant roles in the development of sepsis [5–7]. Plasminogen activator inhibitor-1 (PAI-1), an antifibrinolytic protein, is a member of the serine protease inhibitor (serpin) superfamily that inhibits generation of the key enzyme, plasmin, and subsequent fibrinolysis, by inactivating both tissue-type plasminogen activator (tPA) and urokinase-type plasminogen activator (uPA) [8–10]. Elevated levels of PAI-1 have been observed in patients with severe sepsis, and are commonly associated with unfavorable outcomes and increased mortality [9,11–13], while the reduction of PAI-1 levels is associated with improved survival in patients [13,14].

PAI-1 is not expressed in the kidney but is rapidly induced in a variety of acute and chronic renal diseases, and has been associated with increased fibrin deposition and renal failure [15]. Studies in renal kidney models have shown that PAI-1-deficient (PAI-1^−/−^) mice or mice treated with a PAI-1 inhibitor, deposit less fibrin resulting in less damage to kidneys [16,17], whereas transgenic mice overexpressing PAI-1 develop severe renal fibrin deposition [18,19]. These data suggest that increased PAI-1 levels in kidney play an important role in the pathogenesis of AKI by inducing renal fibrin deposition. It is predicted that the increased renal PAI-1 activity during kidney injury is mediated by vitronectin (Vn), a ∼70 kDa plasma protein that acts as a stabilizing factor of PAI-1 [15,20]. Binding of Vn to PAI-1 increases the half-life of PAI-1, stabilizes the active PAI-1 conformation, and may serve to trap the active PAI-1 within the extracellular matrix [15,20]. However, the *in vivo* role of PAI-1-Vn interaction during sepsis and resultant AKI remains to be established. Moreover, the regulatory mechanisms and signaling pathways by which PAI-1-Vn interaction affects sepsis-induced AKI are not well understood.

In the present study, a LPS-induced model of endotoxemia was utilized in wild-type (WT), PAI-1 deficient (PAI-1^−/−^) mice, and mice expressing a mutant PAI-1 with significantly diminished Vn binding capacity (PAI-1^R101A/Q123K^) [21] in order to gain insights into the role of PAI-1-Vn interaction in response to septic AKI. The results from this study are described herein.

## Materials and Methods

### Mice

Wild-type (WT), PAI-1-deficient (PAI-1^−/−^), and PAI-1^R101A/Q123K^ mice in a C57BL/6J background (males, 8–12 weeks of age) were used in this study and have been described previously [21–23]. Both PAI-1^−/−^ and PAI-1^R101A/Q123K^ mice were visibly normal and showed no apparent developmental defects under non-challenged conditions. Mice were injected, intraperitoneally, with *Escherichia coli* LPS suspended in sterile saline at 10 μg/g body weight (Sigma, St. Louis, MO). At 6 or 24 hr after LPS injection, mice were sacrificed by anesthetizing with rodent cocktail (0.015 mg xylazine/0.075 mg ketamine/0.0025 mg acepromazine/g body weight), and the blood was collected in 4% sodium citrate (1:9 dilution) followed by cardiac perfusion with saline. Kidneys were collected for various analyses described in this study. All experimental animal protocols used in this study (Protocol # 15-002) were approved by the Institutional Animal Care and Use Committee of the University of Notre Dame (Animal Welfare # A3093-01).

### RNA isolation and real time RT-PCR

Renal tissues were homogenized using BD Medimachine System (BD Biosciences, San Jose, CA). Total RNA was extracted using the RNeasy Mini kit (QIAGEN, Valencia, CA). DNA contamination was removed with DNase I treatment. Real time reverse transcription-PCR (RT-PCR) was performed using non-labeled PCR primers and TaqMan probes labeled with 6FAM dye (Eurofins Scientific, Huntsville, AL), as listed in Table 1. PCR reactions were carried out in 96-well plates using ABI 7500 Fast Real-Time PCR system (Applied Biosystems, Foster City, CA). Amplification reactions were performed as follows: 30 min at 50°C and 1 min at 95°C, and 40 cycles of 15 sec at 95°C and 1 min at 60°C. Each sample was run in triplicate. Gene expression levels were expressed as 2^−ΔCT^, in which CT represents the threshold cycle number of RT-PCR at which the amplified product was first detected. ΔCT was determined by the CT of the target gene minus the CT of the reference gene (β-actin).

**Table 1:** List of murine gene specific primers and probes* for RT-PCR.

Gene	Primers	Sequence (5’ to 3’)
β-actin	Forward primer	AGAGGGAAATCGTGCGTGAC
	Reverse primer	CAATAGTGATGACCTGGCCGT
	Probe	CACTGCCGCATCCTCTTCCTCCC
IL-6	Forward primer	CTTCAACCAAGAGGTAAAAGATTTA
	Reverse primer	TAGCCACTCCTTCTGTGACTCC
	Probe	CCTTCCTACCCCAATTTCCAATGCTCTCCTA
TNF-α	Forward primer	CCCCAAAGGGATGAGAAGTTC
	Reverse primer	GCTTGTCACTCGAATTTTGAGAA
	Probe	TCATCAGTTCTATGGCCCAGACCCTCA
MCP-1	Forward primer	GTTGGCTCAGCCAGATGCA
	Reverse primer	AGCCTACTCATTGGGATCATCTTG
	Probe	TTAACGCCCCACTCACCTGCTGCTACT
KC	Forward primer	CTGGGATTCACCTCAAGAACATC
	Reverse primer	CAGGGTCAAGGCAAGCCTC
	Probe	TTGCCCTCAGGGCCCCACTG
ICAM-1	Forward primer	GGAGGTGGCGGGAAAGTT
	Reverse primer	AGGTCCAGTTCCCCAAGCA
	Probe	CGTGCTGTATGGTCCTCGGCTGGA
PAI-1	Forward primer	GACACCCTCAGCATGTTCATC
	Reverse primer	AGGGTTGCACTAAACATGTCAG
	Probe	TCCTGCCTAAGTTCTCTCTGGAGACTGAAG
IL-10	Forward primer	GGTGTCCTTTCAATTGCTCTCAT
	Reverse primer	ATCAAAGGATCTCCCTGGTTTCT
	Probe	ACCCATGAGTTTCTTCACAACTCTCTTAGGAGCT

*The probe was labeled at the 5’-end with the reporter dye, 6FAM, and at the 3’-end with the quencher dye, BHQ1a (Eurofins Scientific, Huntsville, AL).

### Histology and Immunohistochemistry

The harvested kidney specimens were fixed in 4% paraformaldehyde, paraffin processed, and embedded. For general morphological observations, 3 μm sections were cut and stained with hematoxylin & eosin. The specimens were sectioned at 4 μm for immunohistochemical studies. Endogenous peroxidase activity was quenched with 0.3% hydrogen peroxide, followed by microwave exposure in 10 mM citric acid for antigen retrieval. Sections were blocked with 20% normal donkey serum (Jackson ImmunoResearch, West Grove, PA) and incubated overnight at 4° C with goat-anti-mouse fibrinogen (Nordic Immunological Laboratories, The Netherlands) or polyclonal rabbit-anti-mouse PAI-1 (ABCAM, Cambridge, MA) antibodies. Sections were labeled with an AffiniPure peroxidase-conjugated donkey-anti-goat or donkey-anti-rabbit F(ab’)_2_ IgG (Jackson ImmunoResearch). DAB (diaminobenzidine) (Vector Laboratories, Inc, Burlingame, CA) was then applied for color development, followed by Hematoxylin QS nuclear counterstain (Vector Laboratories, Inc.). The slides were mounted, digitally scanned at 40X magnification, and analyzed with an Aperio Technologies CS Digital Slide Scanner (Leica Biosystems, Inc., Buffalo Grove, IL).

The kidney injury score was determined using a semiquantitative score for tubular injury as previously described [24]. Briefly, severity scale values were assigned a score ranging from 0 to 3 (0 = normal, 1 = mild, 2 = moderate, and 3 = severe pathology) for each of 3 variables: tubular vacuolization, tubular dilatation, and tubular cast formation. More specifically, for each variable within each field, a score of 0 was assigned when <5% of the renal tubules were affected, a score of 1 for 5–33%, a score of 2 for 34–66%, and a score of 3 when >66% tubules were affected. For each kidney sample, nine fields (magnification: 40x) were examined at random and the scorer was blinded to the genotype and experimental conditions.

### ELISA and SCr Assays

Plasma concentrations of cytokines and chemokines were determined by a murine ELISA kit (RayBiotech, Inc., Norcross, GA) for TNF-α, IL-6, MCP-1, KC, and IL-10. D-dimer was determined using a D-dimer ELISA kit as previously described (Diagnostica Stago) [25]. The SCr level was measured with the VetTest Chemistry Analyzer (IDEXX Laboratories, Atlanta, GA).

### Western blotting

Protein extracts from renal tissues were prepared by homogenization with glass tissue grinders (Kimble Chase, Rockwood, TN) in 1X cell lysis buffer (Cell Signaling, Danvers, MA). Samples were mixed with 1X Laemmli sample buffer, boiled, separated on a SDS-PAGE gel, and then transferred to PVDF membranes. Membranes were blocked with 5% nonfat dry milk, and then incubated overnight at 4° C with primary antibodies. Membranes were washed with TBST buffer (50 mM Tris, pH 7.4, 0.15 NaCl, and 0.05% Tween 20) and then incubated with horseradish peroxidase (HRP)-conjugated secondary antibody. Protein bands were visualized using the Clarity Western ECL kit (Bio-Rad, Hercules, CA) in a ChemiDoc MP system (Bio-Rad). The primary antibodies used were: monoclonal mouse-anti-β-actin (GenScript, Piscataway, NJ), polyclonal goat-anti-mouse ICAM-1 (R&D Systems, Minneapolis, MN), polyclonal rabbit-anti-rat ERK1/2 and polyclonal rabbit-anti-human phospho-ERK1/2 (Thr202/Tyr204) (Cell Signaling), polyclonal sheep-anti-mouse PC (Haematologic Technologies, Essex Junction, VT), and polyclonal rabbit-anti-mouse PAI-1 (ABCAM). The HRP-conjugated secondary antibodies used were: horse-anti-mouse IgG and goat-anti-rabbit IgG (Cell Signaling), donkey-anti-goat IgG (Jackson ImmunoResearch), and donkey-anti-sheep IgG (AbD Serotec, Raleigh, NC).

### Protein purification and aPC measurements

Cloning, expression, and purification of recombinant mouse PC were performed as follows. The cDNA corresponding to full-length mouse PC was inserted into the eukaryotic expression vector pcDNA3.3-TOPO (Life Technologies). The integrity of the construct was verified by DNA sequencing analysis. HEK 293 cells were transfected with the plasmid using Lipofectamine 2000 reagent (Life Technologies). Transfected cells were grown in Dulbecco's modified Eagle's medium (DMEM) supplemented with 10% fetal bovine serum, 1% antibiotics solution (Sigma), and 200 μg/mL Geneticin (Life Technologies). After 2–3 weeks, high PC expressing colonies were identified by Western blotting as described above using a monoclonal rat-anti-mouse PC antibody (Haematologic Technologies). The selected clones were maintained in the same media containing 50 μg/mL Geneticin. Purification of PC zymogen and its conversion to aPC were performed as previously described [26,27]. The enzyme activity of aPC at 405 nm was determined by using a specific substrate for aPC, SaPC-21 (Aniara, West Chester, OH). The purity of the protein was assessed by SDS-PAGE.

A new approach measuring aPC levels in mouse plasma was developed using anion-exchange Q Sepharose Fast Flow (FFQ) resin (GE Healthcare, Pittsburgh, PA). For this purpose, citrated mouse blood was collected into tubes containing 20 mM benzamidine. Plasma was prepared by centrifuging blood at 14,000 rpm for 15 min. Plasma was incubated with resin pre-equilibrated with a buffer (20 mM Tris, 0.15 M NaCl, 5 mM benzamidine, 2 mM EDTA, pH 7.4) at 4° C for 4 h. After incubation, the anion exchange resin was washed with a buffer (20 mM Tris, 0.15 M NaCl, 5 mM benzamidine, pH 7.4), followed by two additional washes with TBS buffer (20 mM Tris, 0.15 M NaCl, pH 7.4). Finally, the resin-bound proteins were eluted in elution buffer (20 mM Tris, 0.5 M NaCl, pH 7.4). The aPC activity in the eluted samples was determined as described above. The amount of aPC was calculated using a standard curve generated from a known amount of recombinant mouse aPC. Each experiment was performed in triplicate in a 96-well plate.

### Statistical analysis

The significance of experimental differences was evaluated by Student *t* tests (GraphPad Software, Inc; La Jolla, CA) with the level of significance set at *P*≤0.05.

## Results

### Abrogation of PAI-1-Vn interaction protects mice against LPS-induced severe AKI and renal apoptosis

The function of PAI-1 in renal pathology is likely dependent on the high-affinity binding of PAI-1 to Vn, which may facilitate the accumulation of active PAI-1 at sites of renal injury. In order to understand the *in vivo* role of the PAI-1-Vn interaction in septic AKI, PAI-1^R101A/Q123K^ mice that express a PAI-1-mutant in which the interaction between PAI-1 and Vn is selectively abrogated while other functions are retained, were utilized in these studies [21]. In addition, WT and PAI-1^−/−^ mice were also employed for comparison. These mice were then subjected to LPS-induced endotoxemia. It was recently demonstrated that both PAI-1^−/−^ and PAI-1^R101A/Q123K^ mice have reduced mortality compared to WT mice in a LPS-induced mouse model of endotoxemia [21].

**Fig 1:**
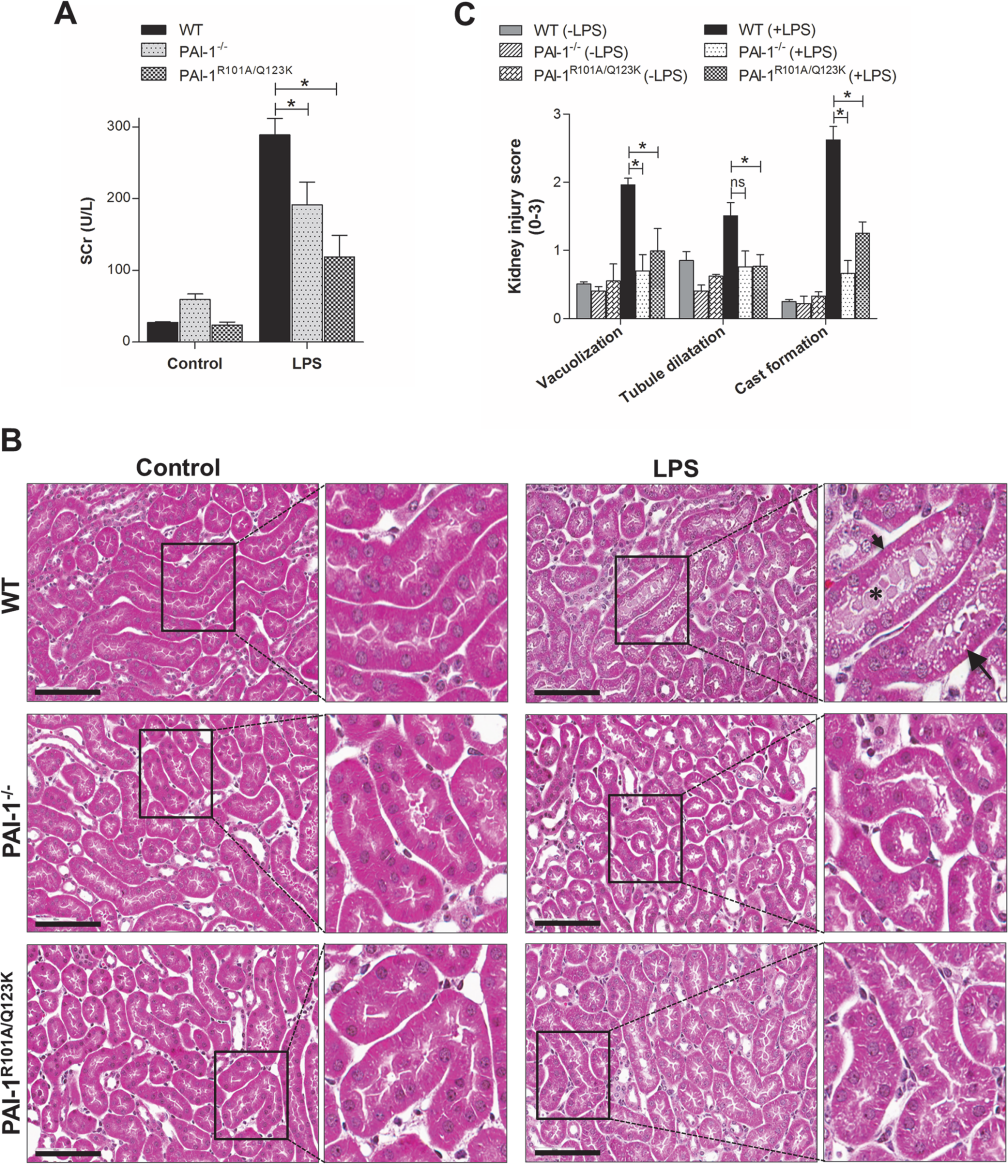
Assessment of kidney injury after LPS challenge. (A) Male WT, PAI-1^−/−^, and PAI-1^R101A/Q123K^ mice were assessed for kidney injury by measuring SCr levels after 24 hr LPS challenge as described. Values are expressed as mean ± SEM (N = 3–5 mice per group), *P<0.05. (B) A representative mouse renal histological image 24 hr after LPS injection. Kidney tissues were stained with H&E as described. Vacuolization (long arrow), cast formation (asterisk), and dilitated tubules (small arrow), are indicated. Magnification: 40x (scale bar, 100 μm). (C) Semiquantitative analyses of renal injury score in LPS-challenged mice as described. Data are expressed as mean ± SEM (N = 3 mice per group), *P<0.05. ns indicates not significant.

Initially, renal function in mice was determined by measuring the serum creatinine (SCr) levels after a single injection of LPS (10 μg/g). As expected, SCr levels increased in the blood of all mice after LPS-challenge indicating the onset and progression of septic AKI (Fig. 1A) [28]. However, PAI-1^−/−^ and PAI-1^R101A/Q123K^ mice showed significant reduced levels of SCr compared with WT mice. The analysis of other well known kidney injury parameters BUN (Blood Urea Nitrogen) and proteinuria were not determined since mice injected with LPS undergo severe dehydration and anuria that generally cause levels of BUN to artificially rise in blood and significant loss of urine production and excretion [29,30].

In addition to diminished renal function, LPS treatment has been shown to induce renal tubular injury (most prominent in the renal cortex) [24, 30]. To determine whether PAI-1^−/−^ and PAI-1^R101A/Q123K^ mice are protected against pathologic injury after LPS challenge, kidney tissue sections were stained with hematoxylin and eosin (H&E). Histopathological examination showed that both PAI-1^−/−^ and PAI-1^R101A/Q123K^ mice have reduced kidney damage, as revealed by diminished tubular vacuolization and degeneration, and cast formation (Fig. 1B and C).

**Fig 2:**
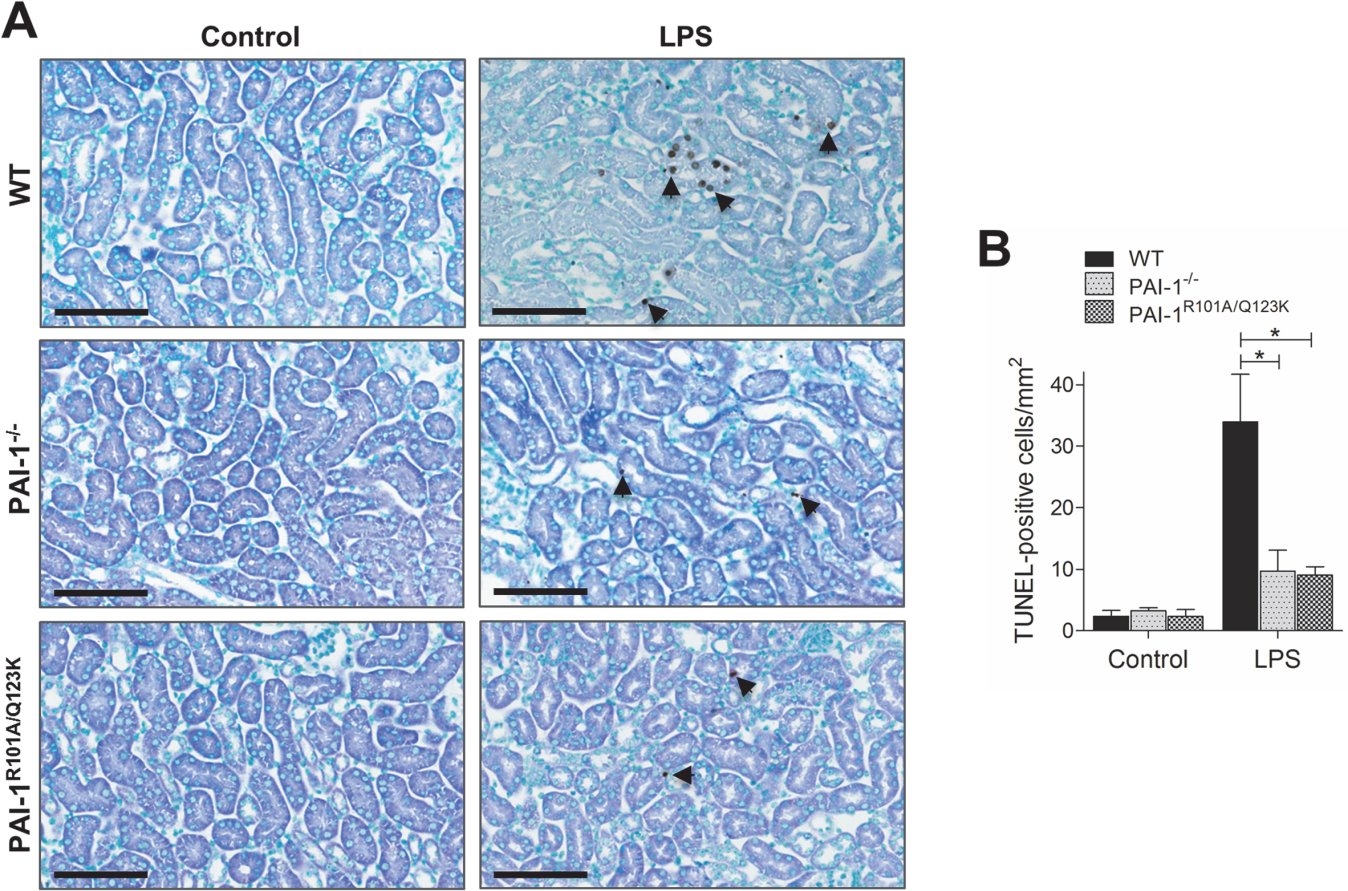
Apoptosis in kidney tissue 24 hr after LPS challenge. (A) A representative terminal deoxynucleotidyl transferase (TdT)-mediated dUTP nick end labeling (TUNEL) staining in the mouse kidneys using the QIA33 FragEL DNA Fragmentation Detection Kit (EMD Millipore, Billerica, MA). TUNEL-positive nuclei (dark brown) in representative kidney sections are indicated by arrows. Magnification: 40x (scale bar, 100 μm). (B) The panel represents the percentage of TUNEL-positive nuclei from different groups. The TUNEL-positive nuclei in nine fields (500 μm^2^/field) from three separate sections of each kidney sample were counted and plotted as the average number of nuclei per mm^2^. Data are expressed as mean ± SEM (N = 3 mice per group), *P<0.05.

Apoptosis is known to play an important role in septic AKI [31]. To evaluate the role of PAI-1-Vn interaction in renal apoptosis, we examined apoptosis in the renal tissue of LPS-challenged mice by TUNEL assays. Histological examination of the kidneys showed enhanced apoptosis in LPS-challenged WT mice relative to PAI-1^−/−^ and PAI-1^R101A/Q123K^ mice (Fig. 2A and B). Thus, a PAI-1 deficiency, or lack of its Vn binding capacity, conferred protection against LPS-induced morphological and functional injury, as well as apoptosis in kidney.

### Selective loss of the PAI-1-Vn interaction decreases LPS-induced renal inflammatory responses

**Fig 3:**
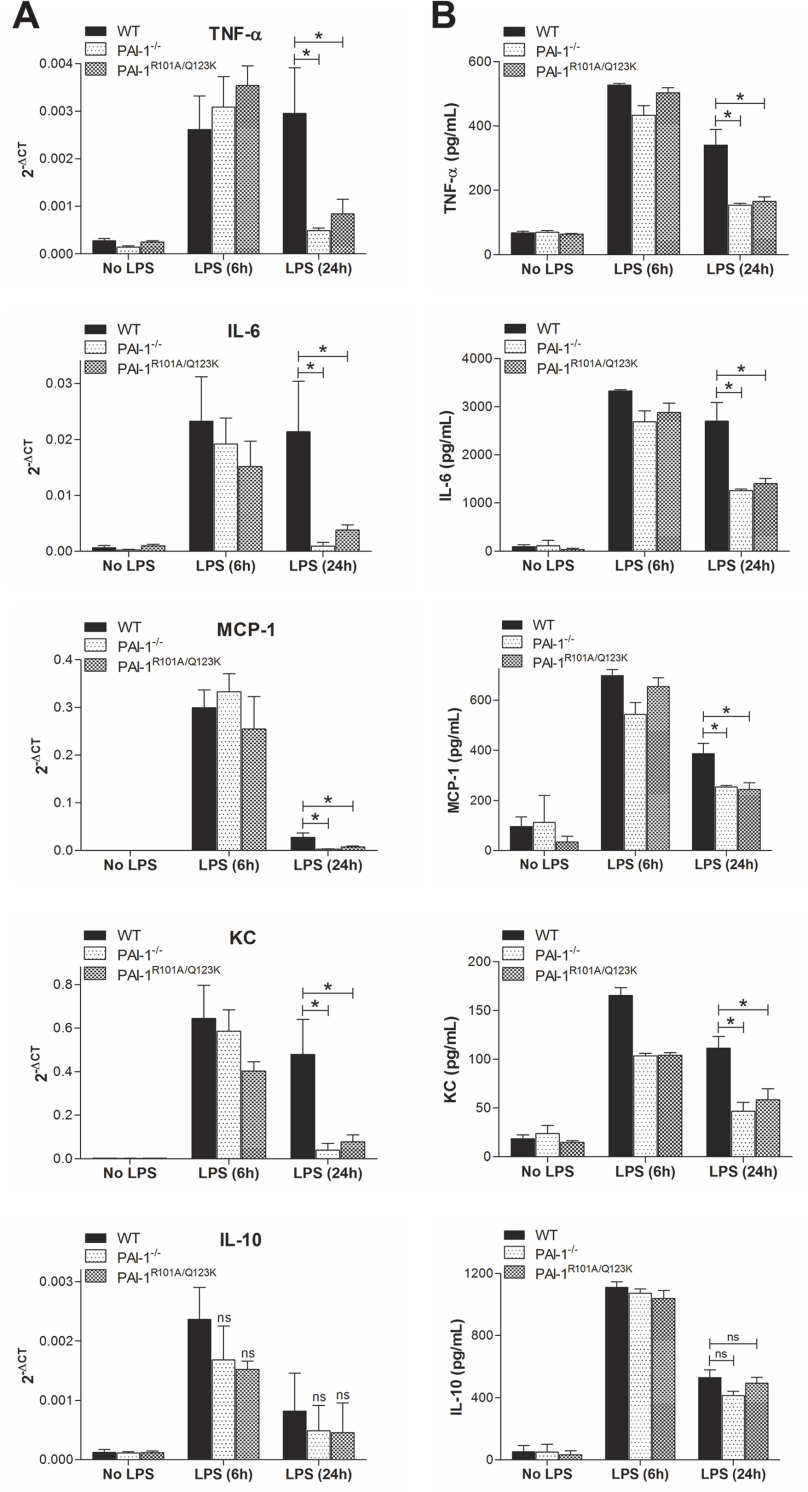
Loss of PAI-1-Vn interaction decreases proinflammatory cytokine and chemokine production after LPS challenge. Mice were challenged with LPS for 6 and 24 hr. (A) Real-time RT-PCR analysis of renal cytokine and chemokine mRNA. Data are expressed as mean ± SEM (N = 6–7 mice per group), *P<0.05. (B) ELISA assays of plasma levels of cytokine and chemokine in mice after LPS challenge. Values shown are mean ± SEM (N = 5 mice per group), *P<0.05.

Inflammation is currently believed to play a major role in the pathogenesis of AKI resulting from sepsis [32]. It is hypothesized that sepsis initially induces functional and morphological changes in renal tubular and vascular endothelial cells leading to the infiltration of leukocytes into the injured renal tissues. Renal injury also induces upregulation and release of proinflammatory mediators, such as cytokines and chemokines, by renal tubular and endothelial cells which contribute to the recruitment of leukocytes into the renal tissues [30,32–34]. Therefore, cytokines and chemokines produced in kidney under pathological conditions are important for both the initiation and progression of inflammation and lead to AKI. For example, overexpression of renal tumor necrosis factor-α (TNF-α) during septic AKI can induce cytotoxicity in renal cells [35]. Studies of other cytokines and chemokines, including interleukin-6 (IL-6), monocyte chemotactic protein-1 (MCP-1), and keratinocyte chemoattractant (KC), were shown to be increased in kidney during endotoxemia and renal inflammation [30,34]. On the other hand, Interleukin-10 (IL-10), a potent anti-inflammatory cytokine, was shown to inhibit inflammatory and cytotoxic pathways implicated in AKI and therefore provides an adaptive mechanism to balance the increased proinflammatory responses [32]. As expected, 24 hr after injection of LPS into WT mice, upregulation of both IL-10 and proinflammatory markers in kidney tissues was observed (Fig. 3A). However, the expression of proinflammatory markers was significantly decreased in both PAI-1^−/−^ and PAI-1^R101A/Q123K^ mice as compared to WT mice after LPS-challenge (Fig. 3A). On the contrary, no significant differences in renal IL-10 expression were observed in all genotypes. At an earlier time point (6 hr), renal cytokines (TNF-α, IL-6, MCP-1, and KC) were equivalently increased in all genotypes, but were only sustained in WT mice at 24 hr. As expected, similar results were observed in plasma levels of cytokines and chemokines in mice after LPS challenge (Fig. 3B). Taken together, these data indicate that loss of PAI-1 or selective attenuation of the PAI-1-Vn interaction suppresses sustained expression of inflammatory cytokines and chemokines in kidneys of LPS-challenged mice.

**Fig 4:**
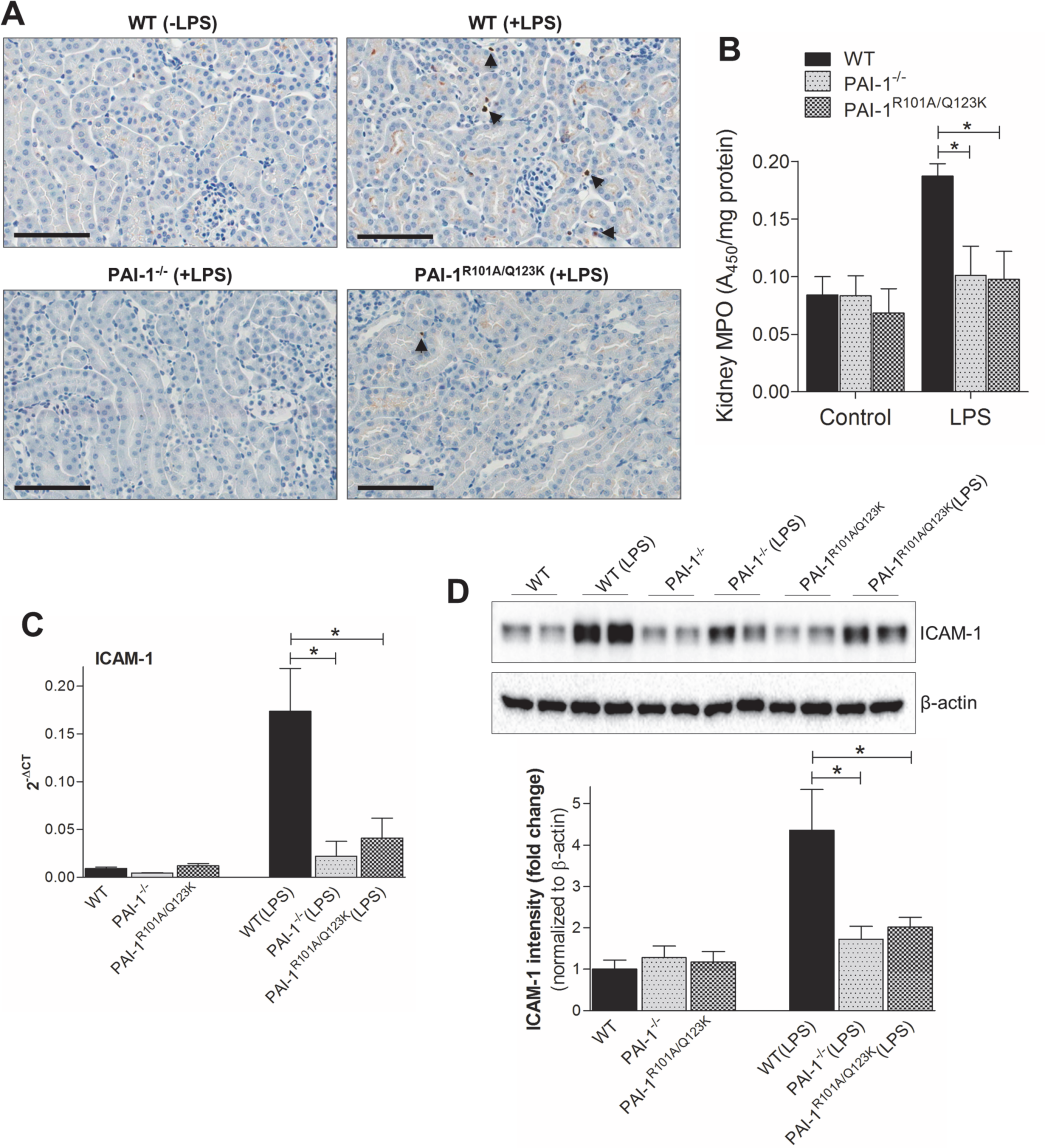
Neutrophil infiltration and ICAM-1 expression in kidney 24 hr after LPS challenge. (A) Immunolocalization of neutrophils (indicated by arrows) in mice kidneys using anti-NIMP-R14 antibody (Abcam). Magnification: 40x (scale bar, 100 μm). (B) Neutrophil infiltration was assessed by measuring renal MPO activity as previously described [37,38]. Data are expressed as mean ± SEM (N = 3 mice per group), *P<0.05. Real time RT-PCR (C) and Western blot (D) analysis of ICAM-1 expression in renal tissues after LPS challenge. Western blot analysis was performed using anti-ICAM-1 and anti-β-actin antibody as described. Densitometric analysis of relative ICAM-1 expression after normalization with β-actin is shown in the lower panel. Data are expressed as the mean ± SEM (N = 5–6 mice per group), *P<0.05.

LPS administration in mice induced infiltration of leukocytes, such as neutrophils, into injured kidneys [32,36]. The extent of neutrophil infiltration in the kidneys was determined by both immunohistochemistry and measuring the activity of the neutrophil-specific enzyme, myeloperoxidase (MPO) [37,38]. As reported earlier, LPS injection caused a significant infiltration of neutrophils in kidney (Fig. 4A). In contrast to WT mice, PAI-1^−/−^ and PAI-1^R101A/Q123K^ mice had significantly fewer neutrophils within the kidney after LPS challenge. Similarly, the MPO activity in renal tissues was lower in both LPS-challenged PAI-1^*−/−*^ and PAI-1^R101A/Q123K^ mice as compared with challenged WT mice (Fig. 4B). These data indicate that PAI-1 deficiency or loss of PAI-1-Vn interaction results in lower neutrophil infiltration into the kidneys of LPS-challenged mice. Renal injury also stimulated expression of adhesion molecules by vascular endothelial cells, such as vascular cell adhesion molecule-1 (VCAM-1), intercellular adhesion molecule-1 (ICAM-1), and P-selectin [36]. These adhesion molecules promote interaction of leukocytes with the endothelium, leading to accumulation of leukocytes around injured sites in kidneys. To determine whether the absence of PAI-1 or the PAI-1-Vn interaction is capable of inhibiting adhesion molecule expression, the expression of ICAM-1 in kidneys was determined by both real-time RT-PCR and western blotting. As shown in Fig. 4C and D, ICAM-1 mRNA and protein expressions were significantly lower in the PAI-1^*−/−*^ and PAI-1^R101A/Q123K^ mice than WT mice following LPS challenge.

### LPS-induced ERK signaling is repressed in PAI-1^−/−^ and PAI-1^R101A/Q123K^ mice

**Fig 5:**
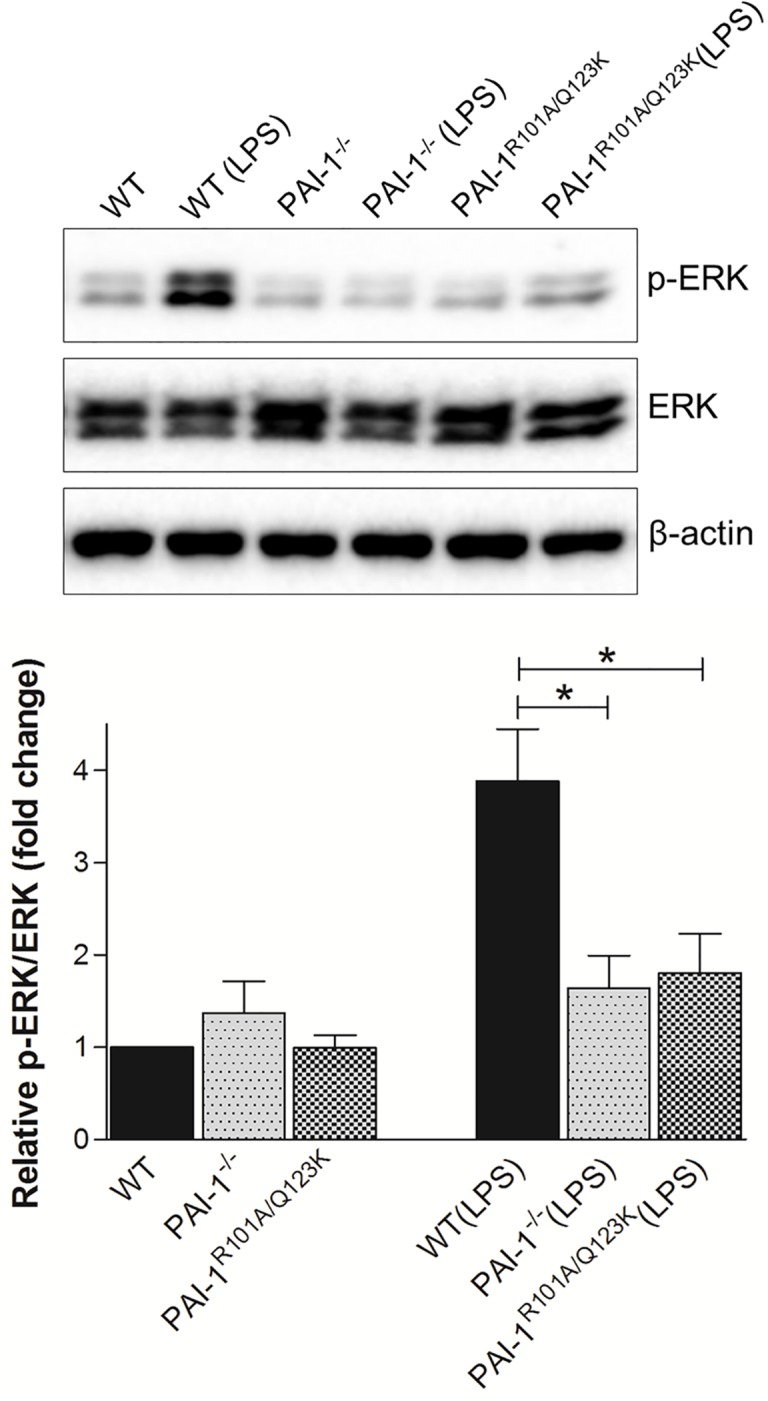
LPS-induced ERK signaling in kidneys 24 hr after LPS challenge. Protein extracts from renal tissues were prepared as described, and then subjected to Western blot analysis of total ERK1/2 (ERK) and phosphorylated ERK1/2 (p-ERK). β-actin was used as a loading control. The relative p-ERK expression was determined by densitometric analysis (lower panel). Data are expressed as the mean ± SEM (N = 5–6 mice per group), *P<0.05.

Renal injury is mediated by a number of factors, including cytokines, chemokines, growth factors, and metabolic toxins, *via* multiple mechanisms and signaling pathways. Among these, the extracellular signal-regulated kinase (ERK) has been identified as an important signaling pathway in which activation results in tissue injury in the kidney [39,40]. To determine ERK activation in renal tissue from LPS-challenged mice, western blot analysis was performed using a phospho-specific ERK antibody. As expected, LPS challenge dramatically increased active phosphorylated ERK (p-ERK) levels in WT mice at 24 hr (Fig. 5). However, the level of p-ERK in both PAI-1^−/−^ and PAI-1^R101A/Q123K^ mice was significantly diminished compared to WT mice indicating that loss of PAI-1 or abrogation of PAI-1-Vn interaction represses ERK signaling activation after LPS challenge.

### Renal Fibrin deposition is significantly altered in the absence of PAI-1 or PAI-1-Vn interaction

**Fig 6:**
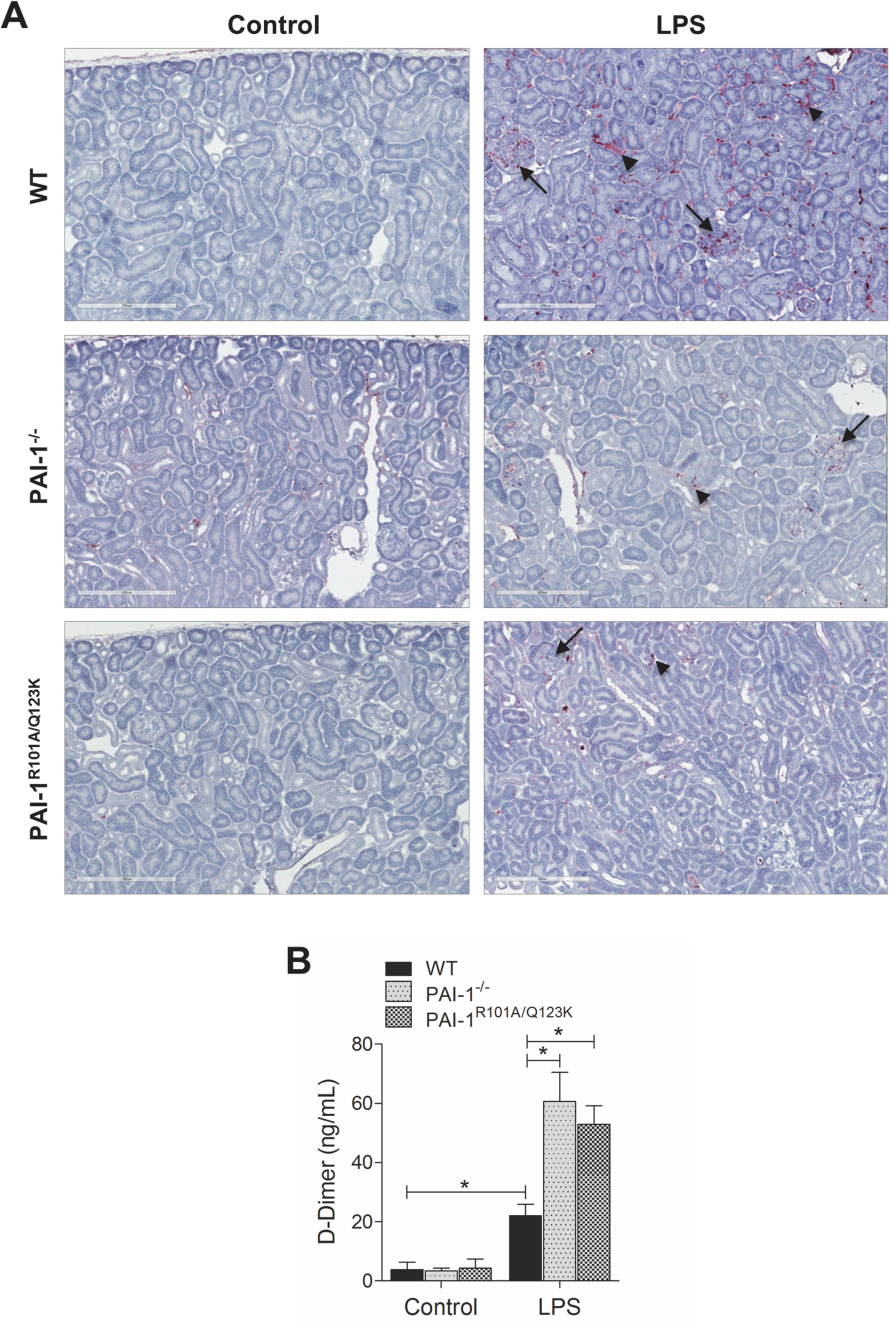
Fibrin deposition in the kidneys 24 hr after LPS challenge in mice. (A) Fibrin deposition in the kidney. Kidney specimens were subjected to immunohistochemical analysis. Fibrin deposits (purple) in both glomeruli (long arrows) and tubules (small arrows), are indicated. Magnification: 20x. (B) Plasma levels of D-dimer. Data are expressed as the mean ± SEM (N = 4 mice per group), *P<0.05.

Sepsis is often associated with a marked increase in fibrin deposition in various organs and tissues [6,7,16]. Fibrin deposition is a common pathological feature in various AKI models and is thought to be one of the important mechanisms of renal damage during sepsis [16,17,41]. As expected, WT mice developed severe fibrin deposition in renal tissues after LPS challenge (Fig. 6A). However, both PAI-1^−/−^ and PAI-1^R101A/Q123K^ mice showed less fibrin deposition. Plasma levels of D-dimer, the degradation product of fibrin, which is commonly elevated in plasma after LPS infusion were also measured [25,42]. As expected, a LPS challenge increased D-dimer levels ∼5 fold in WT mice (Fig. 6B). However, in both PAI-1^−/−^ and PAI-1^R101A/Q123K^ mice D-dimer levels were significantly enhanced relative to WT mice after LPS challenge suggesting that fibrinolysis is stimulated in the absence of functional PAI-1 during LPS challenge.

### Loss of PAI-1-Vn interaction is associated with reduced PAI-1 accumulation in kidney

**Fig 7:**
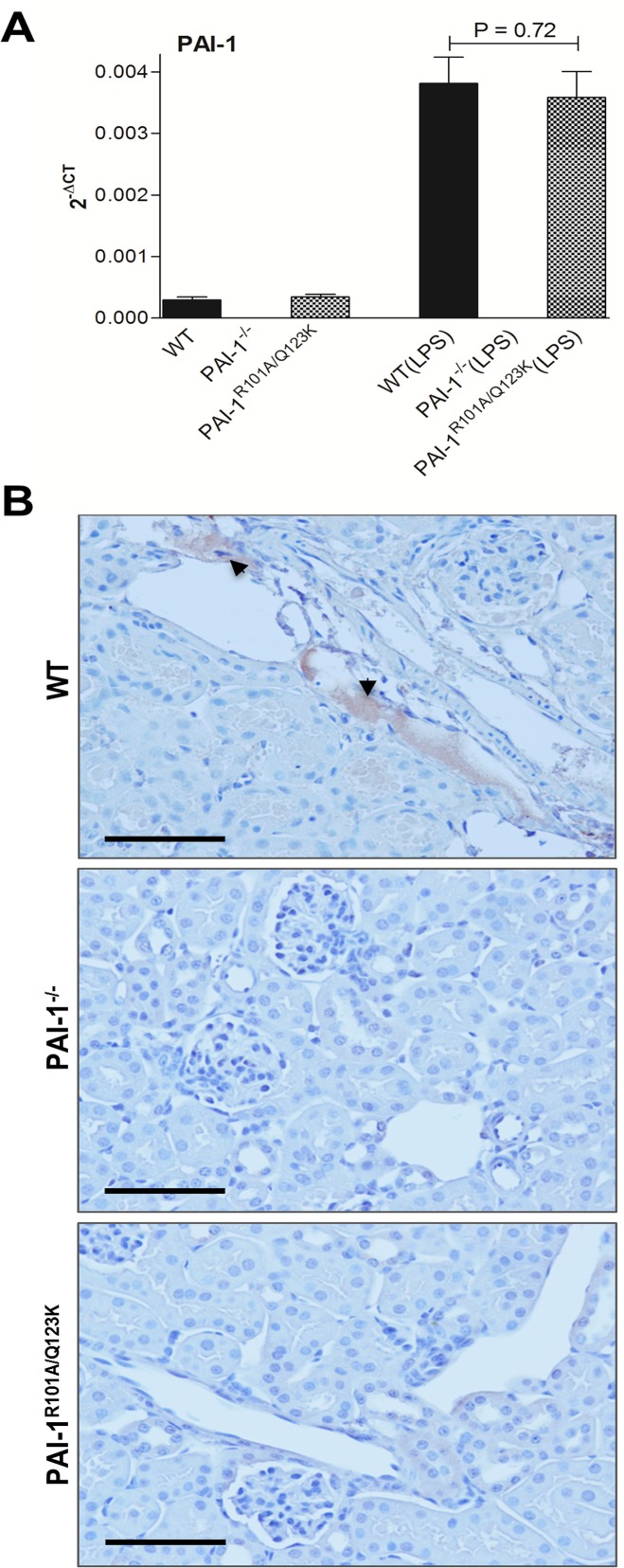
PAI-1 accumulation in the kidney of mice 24 hr after LPS challenge. (A) Real-time RT-PCR analysis of renal PAI-1 mRNA expression. The mRNA expression was determined as described. Data are expressed as the mean ± SEM, (N = 4 mice per group). (B) Immunohistological staining of PAI-1 (reddish brown) in LPS-treated kidney tissues. The experiment was performed as described using a polyclonal anti-mouse PAI-1 antibody. PAI-1 staining in representative micrograph from each group is shown by arrows. Magnification: 40x (scale bar, 100 μm).

Previous studies suggested that increased PAI-1 activity in kidney during injury is mediated by Vn [15]. Vn localizes and functionally concentrates PAI-1 activity in the inflamed kidney (acute glomerulonephritis model), and locally prevents clearance of fibrin [43]. In that study, using a mouse model of acute glomerulonephritis, VN^−/−^ mice demonstrated significantly reduced renal fibrin deposition relative to similarly challenged WT mice. Further, using the model of experimental glomerulonephritis in rats, Huang and colleagues showed that administration of a mutant human PAI-1 (PAI-1R) that binds to Vn normally but does not inhibit plasminogen activators, conferred protection against glomerulonephritis, while a PAI-1 mutant (PAI-1K) that has defective Vn binding but inhibits proteases normally, does not bring any benefit [44,45]. It has previously been demonstrated that both WT and PAI-1^R101A/Q123K^ mice have similar levels of PAI-1 in plasma after LPS treatment [21]. In this study, it was found that these mice express similar levels of PAI-1 mRNA in renal tissue after LPS challenge (Fig. 7A). However, immunohistological analysis demonstrated significant accumulation of PAI-1 in renal tissue of WT mice relative to PAI-1 deposition in PAI-1^R101A/Q123K^ mice after LPS challenge (Fig. 7B).

### Lack of PAI-1 or PAI-1-Vn interaction enhances plasma aPC activity after LPS challenge

**Fig 8:**
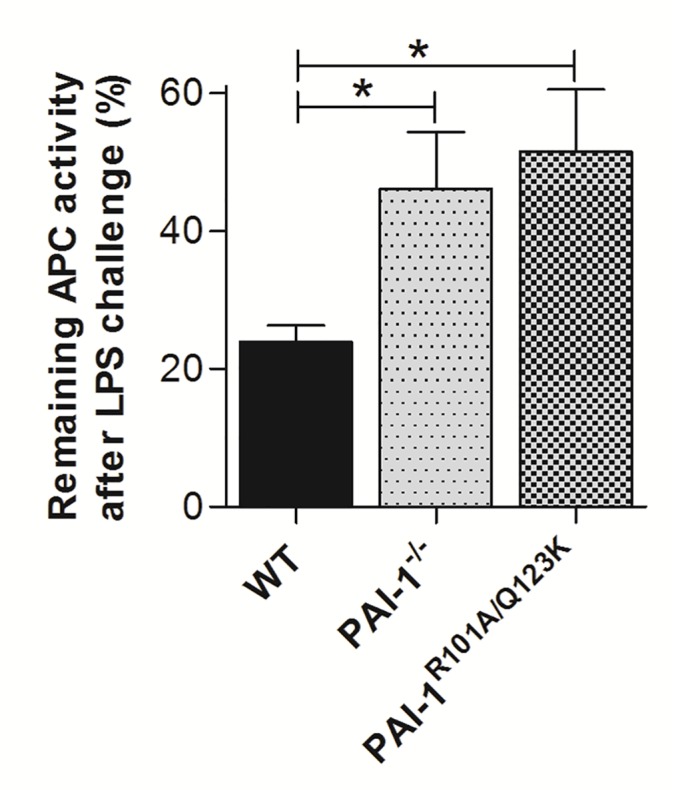
aPC activity in LPS-challenged mice. Citrated plasma was collected from WT, PAI-1^−/−^, and PAI-1^R101A/Q123K^ mice at 24 hr after saline (control) or LPS injection. Plasma levels of aPC were determined as described. Data were plotted as the percentage of aPC activity remaining after LPS challenge, taking the non-LPS control as 100% activity. Data are expressed as the mean ± SEM of 5–6 mice. *P<0.05.

In sepsis, both anticoagulant and fibrinolytic pathways are downregulated. Previous *in vitro* studies demonstrated that human PAI-1 inhibits purified human aPC (a major endogenous anticoagulant protein), and its inhibition is enhanced in the presence of Vn [46,47]. Thus studies were performed to determine whether enhanced survival [21], and protection from renal injury in PAI-1^−/−^ and PAI-1^R101A/Q123K^ mice, after LPS-challenge were the result of increased aPC activity. Plasma aPC activity in non-challenged and LPS-challenged mice was determined. aPC activity in plasma was similar between control groups of mice, but was significantly diminished in LPS-challenged WT mice (Fig. 8). However, both PAI-1^−/−^ and PAI-1^R101A/Q123K^ mice showed significantly higher aPC activity as compared with WT mice, indicating that loss of PAI-1 or PAI-1-Vn interaction protects aPC activity during LPS-induced challenge.

## Discussion

The present study demonstrates that loss of PAI-1 or selective abrogation of the PAI-1-Vn interaction in mice has a protective effect on kidney function, morphology, and cell survival, as compared to WT animals during septic AKI. Therefore, PAI-1 and its interaction with Vn are critical for the development of septic AKI in mice. Most notably, results from this study describe for the first time that the protective effect on kidney functions in the absence of PAI-1 or PAI-1-Vn interaction in mice is also associated with diminished depletion of aPC activity in plasma. This is consistent with previous reports showing that deletion/antagonism of PAI-1 [16–19], or administration of aPC, is associated with decreased renal dysfunction in different AKI models [48,49].

Recent clinical and experimental studies suggest that proinflammatory processes activated during sepsis play pivotal roles in inducing intrarenal hemodynamic changes, endothelial and tubular injury, leukocyte infiltration, and tissue inflammation that ultimately lead to AKI. Bacterial endotoxins, *e*.*g*., LPS, can disseminate to the kidney proximal tubules and microvilli of the brush border [50], where they bind and activate cell surface Toll-like Receptor 4 (TLR4), which results in the downstream activation of ERK signaling [40]. Localized LPS can stimulate the production of key inflammatory mediators, such as TNF-α, by resident tubular epithelial cells [51], glomerular cells [52], and leukocytes adhered to the endothelium [53]. Higher levels of TNF-α are cytotoxic to renal cells and are able to induce injury either directly [35] or through other mechanisms. For example, TNF-α can amplify the induction of localized cytokines and chemokines which stimulates the infiltration of damaging leukocytes into the renal cortex, further amplifying renal inflammation [32,35]. The results of this study showed that the ERK signaling and inflammatory and injury responses in both PAI-1^−/−^ and PAI-1^R101A/Q123K^ mice kidneys after LPS challenge were significantly reduced as compared to WT animals. However, it is unknown if the reduction in ERK signaling and inflammation and injury during LPS-induced AKI are the direct or indirect effect of PAI-1 deficiency or selective loss of PAI-1-Vn interaction.

**Fig 9:**
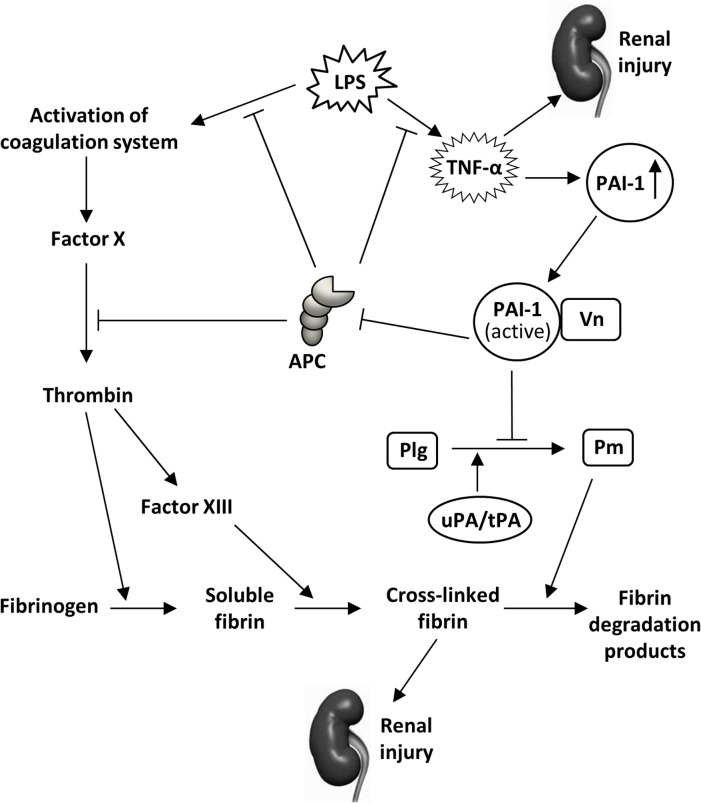
Schematic representation demonstrating functional roles of PAI-1 during LPS-induced AKI. LPS induces AKI in mice by enhancing PAI-1 expression in kidneys. Inhibition of fibrinolysis by PAI-1 through inhibiting tPA/uPA increases fibrin deposition in kidneys. The structural stability, functional activity, half-life, and retention of PAI-1 in renal tissues are increased due to its high affinity binding to Vn [15,20,43]. PAI-1 may also increase severity of LPS-induced AKI in mice by inactivating aPC. Simultaneously, LPS activates the blood coagulation cascade which further results in accumulation of fibrin in renal tissue. Lack of PAI-1 or PAI-1-Vn interaction in kidney leads to decreased renal fibrin deposition and may also rescue aPC function. Increased aPC activity and signaling in kidneys further suppress LPS-induced AKI.

Previous reports suggest that TNF-α and other cytokines mediate LPS-induced increases in PAI-1 expression in several organs including kidney [54]. PAI-1 is a negative regulator of uPA/tPA, essential physiological enzymes involved in fibrin clot and extracellular matrix protein degradation [8–10]. In addition to inflammation, it is believed that the increased levels of renal PAI-1 support fibrin deposition in renal tissues via its antiprotease activity, contributing to AKI (Fig. 9). During this process, the stabilization and localization of PAI-1 at sites of injury are important in inducing maximum renal fibrin deposition. It is speculated that Vn accumulation at sites of renal injury may stabilize PAI-1 and trap active PAI-1 in the vicinity of fibrin deposits [15]. Using a mouse model of acute glomerulonephritis, it has recently been shown that Vn accumulation in the glomeruli can physically localize and functionally concentrate PAI-1 activity in the inflamed glomeruli, and locally prevent the clearance of fibrin [43].

By using a LPS-induced mouse model of endotoxemia in PAI-1^R101A/Q123K^ mice, results from this study demonstrate for the first time that the Vn binding function of PAI-1 is instrumental in septic AKI and most likely stabilizes the inhibitory function of PAI-1 in renal tissue. Specifically, PAI-1^R101A/Q123K^ mice showed similar protection from LPS-induced renal injury as PAI-1^−/−^ mice. We have confirmed that PAI-1^R101A/Q123K^ mice have similar levels of PAI-1 expression in both plasma [21] and in kidney after LPS challenge, and therefore, the protection from renal injury observed in PAI-1^R101A/Q123K^ mice arises from the loss of Vn binding function of PAI-1 and not due to lower PAI-1 levels.

Previous studies demonstrated that LPS challenge in mice induces a rapid drop in plasma aPC activity [55,56]. aPC deficiency associated with AKI can be reversed by aPC treatment in different animal model systems [46,47]. aPC is known to stimulate fibrinolysis by forming a tight 1:1 covalent complex with PAI-1, which leads to the inactivation of PAI-1 [45,57]. At the same time, aPC, in complex with PAI-1, is also not active. The binding interaction between aPC and PAI-1 is relatively slow, but can be increased dramatically by Vn [44,45]. Therefore, it was of interest to determine a possible relationship between septic AKI, aPC, PAI-1, and Vn. It was hypothesized that during sepsis a small fraction of the elevated PAI-1 in the presence of Vn will complex to aPC, and thus lead to depletion of overall aPC activity in plasma and in renal tissues. Consistent with this proposal the current data demonstrated that both PAI-1^−/−^ and PAI-1^R101A/Q123K^ mice have significantly higher aPC activity in plasma as compared with WT mice following LPS challenge, indicating that loss of PAI-1 or PAI-1-Vn interaction inhibits LPS-induced suppression of aPC activity.

In summary, these results have revealed important mechanisms by which PAI-1 drives renal injury during sepsis. The mechanism of this PAI-1 effect in septic AKI is largely dependent on Vn. Therefore, targeting renal specific inhibition of PAI-1-Vn interaction is a potential therapeutic strategy for the treatment of septic AKI.
